# Positive feedback to regional climate enhances African wildfires

**DOI:** 10.1016/j.isci.2023.108533

**Published:** 2023-11-23

**Authors:** Aoxing Zhang, Yuhang Wang, Yufei Zou

**Affiliations:** 1 School of Earth and Atmospheric Sciences, Georgia Institute of Technology, Atlanta, GA, USA; 2 Now at State Environmental Protection Key Laboratory of Integrated Surface Water-Groundwater Pollution Control, School of Environmental Science and Engineering, Southern University of Science and Technology, Shenzhen, Guangdong, China; 3 Now at Our Kettle Inc, Kensington, CA, USA

**Keywords:** Earth sciences, Atmospheric science, Climatology, Atmosphere modelling

## Abstract

Regional climate strongly regulates the occurrence of wildfires partly because drying of fuel load increases fires. The large amounts of aerosols released by wildfires can also strongly affect regional climate. Here we show positive feedback (a seasonal burned area enhancement of 7–17%) due to wildfire aerosol forcing in Africa found in the simulations using the interactive REgion-Specific ecosystem feedback Fire (RESFire) model in the Community Earth System Model (CESM). The positive feedback results partly from the transport of fire aerosols from burning (dry) to wet regions, reducing precipitation and drying fuel load to enhance fires toward the non-burning (wet) region. This internally self-enhanced burning is an important mechanism for the regulation of African ecosystems and for understanding African fire behaviors in a changing climate. A similar mechanism may also help sustain wildfires in other tropical regions.

## Highlights

• The coupled CESM-RESFire model was applied to study fire aerosol feedback in Africa
• Aerosol-climate interactions fuel self-reinforcing Africa wildfires
• Dry-to-wet regional transport of fire aerosols elevates subsequent-season fires

## Graphical abstract

**Figure:**
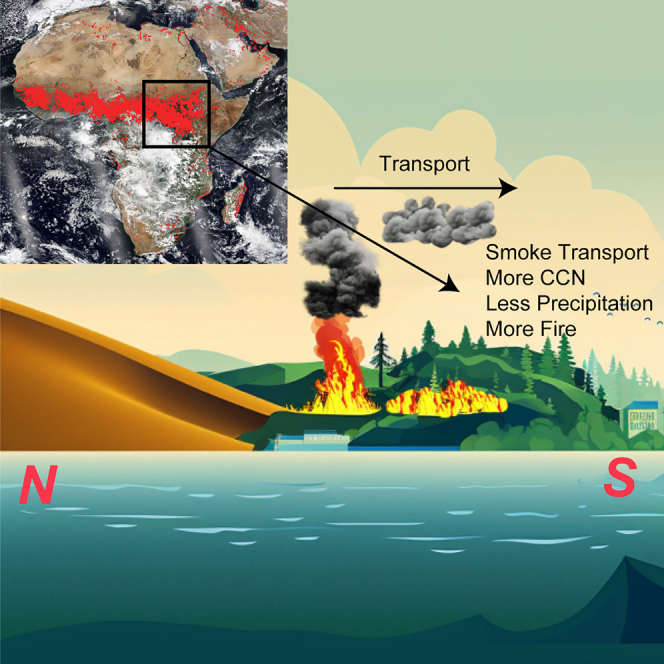

## Introduction

Wildfire initiated by natural or human factors has a profound impact on ecosystems,^1^ carbon cycles,^2^ climate change^3^ and human society.^4^ Although the increase of population and land use conversion tends to suppress fires over most of regions and potentially in the future,^5^^,^^6^^,^^7^^,^^8^^,^^9^^,^^10^^,^^11^^,^^12^^,^^13^^,^^14^ fire weather season length in Africa has increased by up to 40% in Africa in the past four decades, potentially leading shifts in both the geographical distribution and variability of burned areas.^9^^,^^13^^,^^14^ Wildfires also exert a significant impact on regional and global climate systems, as well and ecosystems. This perturbation stems from a complex interplay of short-term and long-term changes in fire weather, terrestrial ecosystems, and human activities. These intricate interactions collectively influence fire intensity, reshaping of fire patterns in various climate-sensitive regions.^15^^,^^16^^,^^17^^,^^18^ While there remain divergent estimations regarding the trends in wildfire emissions across regions,^7^^,^^8^^,^^9^^,^^10^^,^^11^^,^^12^^,^^13^ studies have indicated shifts in both the geographical distribution and variability of burned areas, including those in Africa.^9^^,^^13^^,^^19^

Fires and regional climate interact through several means.^20^ Fires impact climate by reducing local precipitation^21^^,^^22^ and changing the global radiative balance through the emissions of greenhouse gases (GHG) and aerosols.^3^^,^^23^ On the other hand, drought-related fire increase has been found globally.^24^^,^^25^^,^^26^^,^^27^^,^^28^ Climate change has increased the occurrences of weather conditions conducive to fires over the past 40 years,^29^ and the effect will potentially become larger in the future.^30^

Fires are a major source of atmospheric aerosols,^31^ which strongly affect the global radiative balance^2^ and cloud processes.^32^ Aerosols can have both positive and negative impacts on precipitation.^33^ An increase in the number of cloud condensation nuclei (CCN) leads to competition for the available water vapor, resulting in smaller CCN sizes and making it more difficult to form raindrops.^34^ The aerosol radiative impact may also suppress evaporation, which then affects cloud formation. On the other hand, while aerosols may reduce precipitation from shallow clouds, they can stimulate deep convection with a warm cloud base.^35^ A positive feedback among fires, cloud, and precipitation was previously hypothesized.^36^^,^^37^ However, atmospheric modeling studies often focused on the perturbations by fire emissions to regional and global weather and climate,^38^ while fire modeling studies often considered only the impacts of weather and climate on fires.^16^

In this work, we use the interactive Region-Specific ecosystem feedback Fire (RESFire) model in the Community Earth System Model (CESM)^19^^,^^39^ to investigate positive feedback through aerosol forcing that enhances wildfires in Africa, which is an ideal place to study fire and climate interactions. The area of highly flammable savanna-like vegetation dry conditions is vast, and the extensive vegetation cover and favorable weather conditions contribute to substantial fuel accumulation in Africa. As a result, about half of global biomass burning occurs in Africa and the continent has more fire aerosol emissions than any other continent.^40^ The alternation of dry seasons in northern and southern Africa also means that fires are persistent throughout the year in Africa. The environmental and socioeconomic challenges caused by fires are exacerbated by climate change.^41^ Understanding the systematic fire-climate feedback via aerosol forcing in Africa is therefore crucial for examining potential climate change caused vulnerabilities in the continent.

## Results

### Enhanced seasonal to annual variations of fires in equatorial Africa

Africa accounts for >50% of the total area burned by fires on average.^13^^,^^42^^,^^43^ It is also an area with the largest aerosol-cloud radiative forcing by aerosols emitted from fires.^19^ We conduct 10-year RESFire simulations for the present-day climate with and without coupled fire-climate interactions (hereafter referred to as the “fire aerosol feedback” and “no-fire aerosol feedback” simulations, respectively) to explore the impacts and mechanisms of the feedbacks between fire aerosols and regional climate.

Aerosol forcing in general is short-term because the lifetime of aerosols is on the order of 1 week due to wet scavenging.^44^^,^^45^ However, over the equatorial region of Africa (10° S - 10° N, 10° E − 28° E), fire aerosol forcing can lead to variations of the burned area on time scales much longer than a week. Figure S1 compares the power spectral densities (PSDs) of monthly mean burned area over equatorial Africa with and without fire aerosol feedbacks. Aerosol feedbacks enhance the seasonal and annual variations (3, 4, 6 and 12 months) by up to 60%. This simulated fire aerosol feedback differs from those related to fuel load, climate patterns,^46^ or human impact,^47^ and contributes to both short-term (within a week) and long-term (seasonal to annual) fire variations.

### Positive feedbacks of fire aerosols on burning

**Figure 1:**
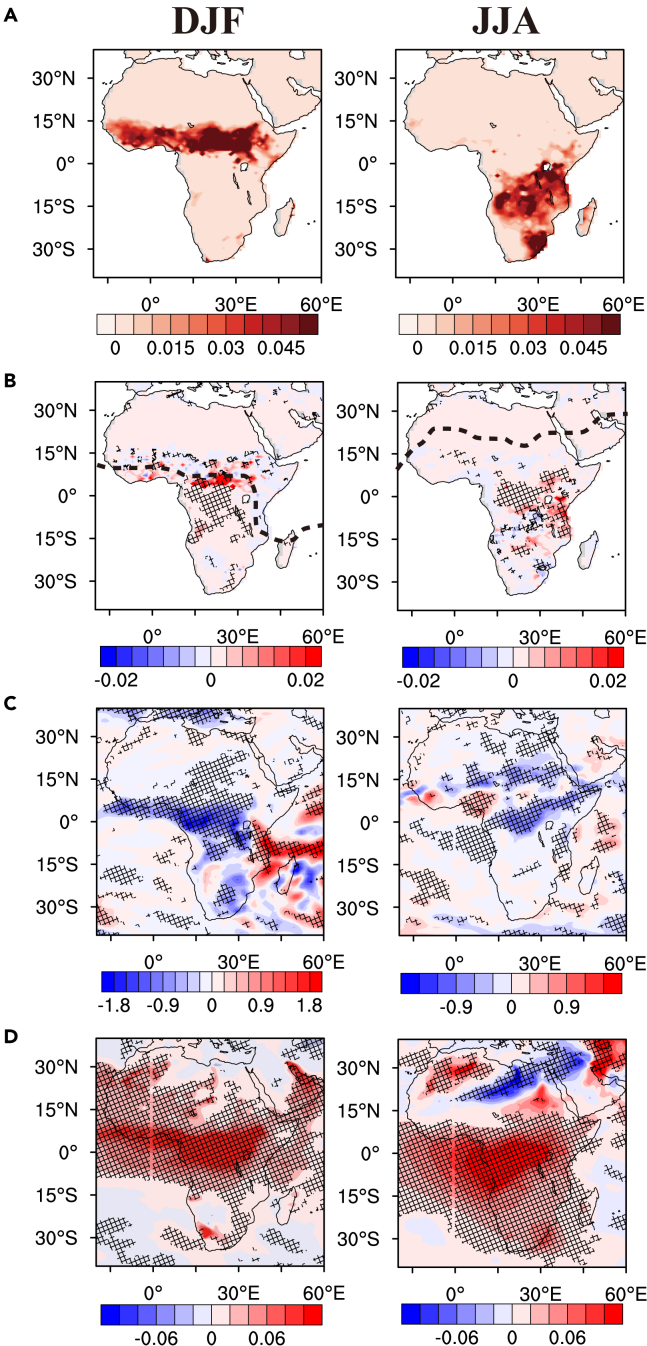
Effects of fire aerosol feedback in Africa in DJF and JJA (A) Burned fraction distribution without fire aerosol feedback. (B) Distribution of burned fraction change caused by fire aerosol feedbacks; the black dashed line denotes the location of ITCZ. (C) Precipitation changes due to fire aerosol feedback (unit: mm/day). (D) Distribution of fire aerosol AOD. In Panels (B), (C), and (D), a t-test was conducted on the seasonal mean values for each gridbox between experiments with and without fire aerosol feedback. Grid boxes with statistically significant differences (p < 0.05) are cross-shaded.

The main fire seasons occur in the Northern Hemisphere (NH) Africa in December, January, and February (DJF) and in the Southern Hemisphere (SH) Africa in June, July, and August (JJA). This seasonal cross-equator fire migration is a key factor for extending short-term aerosol forcing into seasonal and interannual time scales shown in Figure S1. An important clue is shown in Figure 1. The seasonal increase of burned area is 13–25% in equatorial Africa. Compared to NH burning in DJF and SH burning in JJA without fire aerosol feedbacks (Figure 1A), the enhancements of burned area extend from the equatorial region toward the other hemisphere (Figure 1B). The feedback enhancement extends from 0°–8° N to 0°–15° S in DJF and from 0°–30° S to 0°–10° N in JJA, implying the significance of cross-equatorial processes in fire aerosol feedback.

The regional shift of fire aerosol-induced burned area is related to the transport of fire aerosols. The features of wind transport are illustrated by the circulation patterns (Figure S2). While the surface wind pattern around the equator is affected by the location of the intertropical convergence zone (ITCZ), which is mostly to the north of the equator, the wind transport in the lower free troposphere (850 and 700 hPa, 1–3 km in altitude) is quite different. In DJF, the burning takes place in NH Africa (Figure S1). The lofted fire aerosols are transported southward across the equator in the lower free troposphere. In JJA, the burning takes place in the SH Africa. The southerly wind near the surface in part due to the convergence of ITCZ transports fires aerosols northward. Figure S3 shows that the fire aerosol feedback did not significantly change the location of ITCZ, but affected atmospheric circulation. Near the surface, the convergence north to the ITCZ is 10%–20% larger with fire feedback in DJF and MAM, but the increase is less than 10% in JJA and SON. Therefore, the aerosol effects are the major contributor to this feedback mechanism.^19^

As a result of fire-aerosol transport, the distribution of aerosol optical depth (AOD) from fire differs significantly from that of burned areas. The fire aerosol AOD distribution shows that the enhancements shifted more toward the non-burning hemisphere (Figure 1D). The simulation results show that fire aerosols reduce precipitation (Figure 1C). We compared the radiative effects due to direct aerosol-radiative interactions and indirect aerosol-cloud interactions (Figure S4) and find that that the aerosol radiative impact by aerosol-cloud interaction is much larger, in agreement with the previous study.^19^

**Figure 2:**
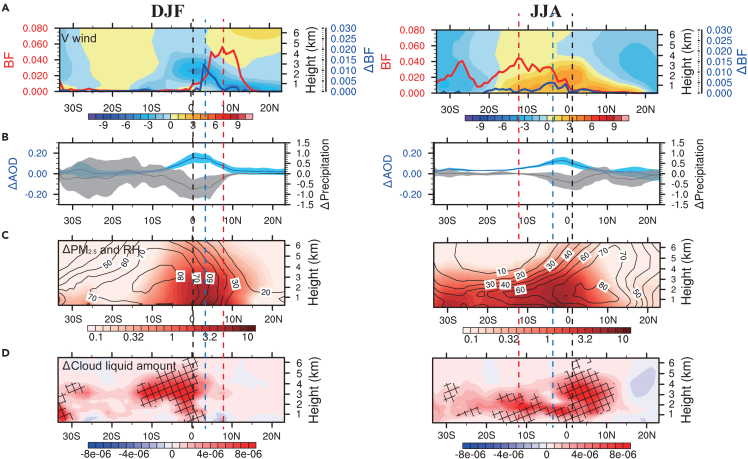
Zonal mean distributions of fire aerosol feedbacks averaged over the African land area in DJF and JJA (A) The burned fraction without fire aerosol feedback (in red lines) and the burned fraction change from no fire aerosol feedback simulation to fire aerosol feedback simulation (in blue lines). Colored contours represent averaged northward-southward wind speed (in unit of m s^−1^, positive values are northward wind). (B) The 15° W - 45° E zonal mean AOD (in blue lines) and precipitation (in black lines, in unit of mm/day) change from the no-fire aerosol feedback simulation to the fire aerosol feedback simulation. The zonal standard deviation from the ensemble mean is shaded. (C) The fire PM_2.5_ distribution (color shaded, in units of μg m^−3^) and the RH change (contour, in units of percentage) from the no fire aerosol feedback simulation to the fire aerosol feedback simulation. (D) The cloud liquid amount (in units of kg kg^−1^) changes from the no fire aerosol feedback simulation to the fire aerosol feedback simulation. A Student’s *t* test was performed to compare the seasonal mean values between experiments with and without fire aerosol feedback. Areas with statistically significant changes (p = 0.05) are cross-shaded. The dashed lines in red denote the latitude of peak burned area; dashed lines in blue denote the latitude of peak burned area change, and the black dashed lines denote the latitude of the most reduced precipitation.

The fire aerosol-cloud effect on precipitation is shown more clearly on the zonal mean distribution changes due fire aerosols (Figure 2). In DJF, the largest zonal-mean burned fraction increase occurs at 2°–8° N. The southward transport of fire aerosols at 1–3 km results in the largest zonal-mean increases of AOD and PM_2.5_ and the largest decrease of precipitation at 0°–4° N, to the south of the largest burned fraction. In-between the former maxima lie the largest zonal-mean increase of burned fraction at 3° N. The largest fire aerosol effect on precipitation is shifted away from the burning region because of the increase of RH and cloud water content away from the burning region. The effect of fire aerosols on precipitation moves toward the burning region for two reasons.^21^^,^^36^ The maximum loading of fire aerosols cools the surface and reduces the vertical instability of the atmosphere. At the edge of the burning region, where RH is relatively low (compared to the rainy region), the influx of fire aerosols competes for the available atmospheric water, increasing the number but reducing the size of CCN, resulting in a reduction of precipitation.^36^ CCN accumulated in the cloud, increasing the amount of low to middle clouds above 1 km (Figure 2D). This could potentially enhance precipitation elsewhere downwind, such as on the East Coast in DJF (Figure 1C), due to the conversion of water vapor into cloud droplets and the convection invigoration mechanism.^33^

Fire aerosol induced drying at the edges of fire regions subsequently increases fires in DJF (Figure 1). The maximum zonal mean burned area increase due to fire aerosols is >50% (Figure 2A), while the maximum relative burned area increase in this region (Figure 1) reached 9 times that without fire aerosol feedbacks. In the non-burning regions in the SH Africa, on the other hand, the reduction of precipitation causes a dryer condition in the subsequent burning season. The observation-based RESFire parameterization predicts larger fire spreads under dryer conditions in the subsequent burning season,^19^^,^^39^ contributing to the increase of burning in March, April, and May (Figure S5). It is worth noting that this effect is inter-seasonal, but its precise quantification in a fully coupled climate model is beyond the scope of this work. The net increase of burned area is a result of both intra-seasonal and inter-seasonal feedbacks of fire aerosols.

Similar fire-aerosol feedback is simulated in JJA, but the seasonal burning takes place in the SH Africa and the positive feedback on burning extends from the northern burning area to the equatorial region with the largest relative zonal-mean enhancement of burned fraction in the equatorial region (Figures 1 and 2). Since the ITCZ, determined by the maximum and zero-gradient latitudes for the surface wind divergence,^48^^,^^49^ is located north of the equator and the burning region, the near-surface flow is northward, bringing large amounts of fire aerosols to the northern equatorial region (Figure S2). At 3–4 km (∼700 hPa), the south-ward flow crosses the ITCZ to 10° N. The flow pattern leads to large fire aerosol loading in the equatorial region, which decreases precipitation near the equator. Since the flow northward-southward direction switch altitude increases from the ITCZ to the equator, the vertical extent of fire aerosols maximizes in the equatorial region (Figure 2). The zonal-mean moisture has a maximum at 10° N, resulting in a larger effect of fire aerosols on cloud liquid amount and precipitation in the northern than southern equatorial region. The maximum increase of the zonal mean burned fraction is 12% of the maximum zonal mean burned fraction. Beyond the intra-seasonal effect, burning moves to the northern equatorial region in September, October, and November (SON). The drying of the northern equatorial region in JJA helps the inter-seasonal burning enhancement in SON.

In DJF and JJA, the largest increases of zonal mean burned fraction, AOD, and cloud liquid water content and the largest decrease of precipitation occur downwind from the largest burning region, where burning occurs in the subsequent season. The DJF and JJA total burned areas are comparable at 2.9 × 10^5^ km/month, larger than 1.2 × 10^5^ km/month in MAM and 1.8 × 10^5^ km/month in SON. The burning in MAM and SON is, however, more widespread covering both NH and SH Africa (Figure S5). The seasonal NH-to-SH shift of precipitation occurs in MAM and the reverse is the case in SON.^50^ Burning occurs in the dry regions shifts seasonally following the shift of the precipitation regions, resulting in burning in both NH and SH Africa simulated in the model similar to the observations^40^ (Figure S5). One consequence of the widespread burning in MAM and SON is that aerosol loading is enhanced by fires, leading to precipitation reduction and burning enhancement in both NH and SH Africa. The geographical spread of burning and aerosol effect result in a larger relative positive feedback by fire aerosols on burning in MAM and SON (15–17%) than DJF and JJA (7–9%), although the absolute burned area increase due to fire aerosols is more comparable among DJF, MAM, JJA, and SON.

## Discussion

Fire aerosols lead to both intra-seasonal and inter-seasonal burned fraction increases (Figures 1 and S5). These increases tend to occur in tropical Africa and can be attributed to the following process. Fire aerosols are transported from the burning (dry) to the non-burning (wet) hemisphere across the equator. A large amount of fire aerosols increases CCN, reduces precipitation,^51^ and increases fire activity.^21^ The intra-seasonal effect occurs on the burning side toward the non-burning region, where a reduction of precipitation provides conducive conditions for more burning. Further down in the non-burning (wet) hemisphere across the equator, drying by fire aerosols in the current season enhances fires in the subsequent burning season. The coupling of the fire-aerosol feedback with the migration of precipitation is more apparent in MAM and SON, resulting in stronger fire-aerosol feedback than in DJF and JJA.

**Figure 3:**
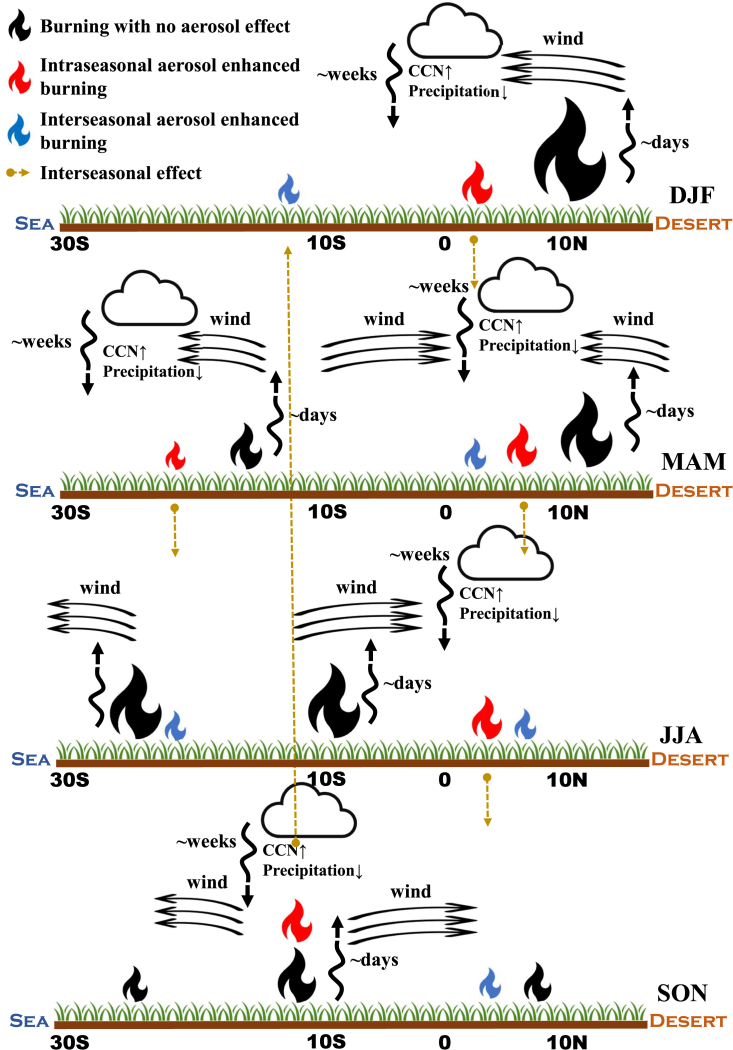
A schematic diagram for the seasonal progression of fire aerosol feedback The colors of fire icons represent burning with no fire aerosol feedback (black), burning of fire aerosol feedback in the same season (red), and burning of fire feedback across the season (blue). The size of fire icons qualitatively represents the burned fractions. The yellow dashed lines point the locations of the inter-seasonal feedback.

Figure 3 shows a schematic diagram of the positive fire-aerosol feedback process. In DJF, the intra-seasonal burning enhancement due to fire aerosols is located at 3.3° N, which is 4.7° south to the peak of the maximum burned fraction. This enhancement from DJF continues to MAM and triggers the burning at the edge of the fire region toward the equator in MAM. The intra-seasonal fire enhancements from local burning in MAM are at 7° N and 16° S, respectively, which continue to JJA. In JJA, fire aerosols in SH Africa are transported near the surface northward and the tropical fire aerosols trigger an enhancement of burning across the equator, which also continues in SON. During the burning migration from the SH to NH in SON, fire aerosols are transported to the north and south of the fire regions, spreading out the burning feedback enhancements in both NH and SH Africa.

### Limitations of the study

The overall effect of the fire aerosol feedback is to widen the region of burning. This positive feedback process simulated by the CESM-RESFire model is difficult to diagnose using the observations only. The good agreement between model simulations and the observations (see the STAR Methods section) lands confidence in the modeling results. However, the fire-aerosol-climate-ecosystem coupling is complex.^39^ As the interactive fire model and aerosol-climate parameterizations improve, additional feedback mechanisms may also exist. For instance, an increase in the intensity of high clouds and deep convection may result in more lightning events. In dry regions with a high potential for wildfires, lightning can play a crucial role in igniting fires.^52^^,^^53^ In addition, the global cooling effect of aerosols may lead to a negative feedback globally in a longer timescale due to a negative correlation between temperature and fire ignition.^53^ The effects of fire aerosols on precipitation vary from region to region.^54^^,^^55^^,^^56^ This process and its effect on burning in Africa, particularly in the context of the seasonal migration of precipitation,^50^ appear to be a key issue for future studies. Although the fire aerosol feedback through wind shift is insignificant in this study, analysis of the fire aerosol impacts to atmospheric circulation on different timescales is still necessary in the future. Furthermore, interactions among aerosols, lightning, and precipitation can also be important for fires in Africa.^57^ We did not consider the feedback from the ocean in this study. It is conceivable that the transport of African fire aerosols to the ocean and the subsequent ocean feedbacks will further increase the climate signals of fire feedbacks.^58^^,^^59^^,^^60^^,^^61^ The timescale of the fire-ocean feedback can easily extend into the multi-decadal timescale.

### Conclusions

The significant spatiotemporal burning and precipitation changes due to fire aerosols (Figures 1 and S6) indicate that the positive fire feedback is an important mechanism for the regulation of African ecosystems. The positive feedback results partly from the transport of fire aerosols from burning (dry) to wet regions, reducing precipitation and drying fuel load to enhance fires toward the non-burning (wet) region. The unique alternation of dry and wet season across the equator in Africa extends the short-term (days to weeks) aerosol forcing into longer inter-seasonal enhancements of burning (Figure 3). The spatiotemporal varying positive feedback of fire aerosols is manifested in the inter-seasonal and intra-annual variability of burning in Africa, increasing the intra-annual PSDs by up to 60% (Figure S1). The multi-year El Niño–Southern Oscillation (ENSO) is not simulated in the model.

The identification of the fire-aerosol positive feedback mechanism in Africa advances our knowledge of climate feedbacks related to wildfires globally. Studies have shown that in some coastal areas (such as the Mediterranean, Southeast Asia, and the western United States), fire smoke alters local fire weather, resulting in positive feedback.^37^ However, these regions have distinct fire seasons, and the local fire intensification caused by this feedback does not persist into the next fire season. In contrast, in Africa, the partial alignment of prevailing winds and shifting fire regions means that the positive feedback from large fire-aerosol events not only occurs within the current fire season but also amplifies burning in the subsequent season. This type of positive feedback implies that a warmer and dryer climate will likely lead to more persistent burning in Africa in the future. The systematic fire-climate feedback may also be present in other fire prone tropical regions and has significant ramifications for understanding the impacts of fires and climate change on humans and plant life.

## STAR★Methods

### Key resources table

**Table:** 

REAGENT or RESOURCE	SOURCE	IDENTIFIER
**Deposited data**		

CESM-RESFire simulations	This paper	ScienceDB: https://doi.org/10.57760/sciencedb.12851
Global Fire Emissions Database (GEFD4.1s)	Giglio et al.^13^; Randerson et al.^42^; Van Der Werf et al.^43^	https://www.globalfiredata.org/
NASA LIS/OTD grid product v2.2	NASA	https://ghrc.nsstc.nasa.gov/uso/ds_docs/lis_climatology/lolrdc_dataset.html
Climate Forecast System Reanalysis	National Centers for Environmental Prediction (NCEP)	https://climatedataguide.ucar.edu/climate-data/climate-forecast-system-reanalysis-cfsr
Global Precipitation Climatology Project Version 2.3	Adler et al.^88^	https://climatedataguide.ucar.edu/climate-data/gpcp-monthly-global-precipitation-climatology-project

**Software and algorithms**		

NCAR Command Language (NCL) 6.6.2	NCAR	https://www.ncl.ucar.edu/

### Resource availability

#### Lead contact

Further information and requests for resources and reagents should be directed to and will be fulfilled by the lead contact, Yuhang Wang (yuhang.wang@eas.gatech.edu).

#### Materials availability

This study did not generate new materials.

#### Data and code availability

The CESM-RESFire simulations generated in this study are available on https://www.scidb.cn/en/s/FruQJf.

### Method details

#### Model description

We make use of the CESM-RESFire model developed in the CESM 1.2 modeling framework.^39^ The RESFire model couples the atmospheric module (the Community Atmosphere Model version 5.3, CAM5^62^ and the land module (the Community Land Model version 4.5, CLM4.5^63^ in the CESM. In this context, the online RESFire model (Figure S6) simulates fire occurrence, spread, and impact as well as the coupling of fires with the atmosphere, vegetation, and land surface.^19^^,^^39^ CAM5 couples the 3-mode modal aerosol module (MAM3)^64^ with a simple secondary organic aerosol (SOA) treatment derived from fixed mass yields of the gas phase SOA precursors,^65^^,^^66^ the online calculation of dust and sea salt aerosols,^62^ the photosynthesis scheme,^67^ the Rapid Radiative Transfer Method for Global Climate Models (RRTMG),^68^^,^^69^ the scheme of cloud macrophysics^70^ and microphysics.^71^^,^^72^ The fire plume rise scheme is parameterized on the basis of the fire radiative power (FRP), planetary boundary layer height (PBLH) and Brunt–Väisälä frequency in the free troposphere.^73^ The CESM-RESFire model considers the regional differences of fires and ecosystems and improves the parameterizations for simulating fire occurrence and spread as functions of weather, climate, ecological, and anthropogenic factors, making it capable to simulate fire emissions and their interactions with climate.^39^ The CESM-RESFire model provides a comprehensive treatment of fire simulations in a climate model and can largely reproduce the observed burning patterns and trends; most importantly, RESFire interfaces between the atmospheric and land models such that the two-way interactions between fire and climate can be quantified.^19^ In a previous study, Zou et al. applied this modeling framework to global fire simulations with a focus on evaluating the model performance with available observations and analyzing the annual mean global fire-climate-vegetation interactions.^19^ In this study, we apply this modeling framework to investigate the seasonal and inter-regional feedback between fire aerosols and regional climate in Africa.

#### Model simulation setup and evaluations

To diagnose and evaluate the impact of the positive fire-aerosol feedback, we ran two 10-year experiments of RESFire model simulations under the present-day scenario (2001-2010), with a model spatial resolution of 0.9°x1.25° and a timestep of 30 minutes. The atmosphere model (CAM) with the fixed SST climatology was spun up for 1 year. In the control run (the “fire aerosol feedback” case), online fire aerosols were simulated using RESFire. In the sensitivity run (the “no-fire aerosol feedback” case), fire aerosols were not included. Different from previous studies on the one-way impact from fire to climate or from climate to fire, this model framework can quantify the impact from the two-way interaction between fire and climate.^19^ Both ensemble simulations share the same initial conditions after a hundred-year spinning-up of CLM-RESFire,^39^ ensuring the attainment of a steady state with regards to land surface type alterations. Furthermore, the RESFire parameterizations were developed to simulate fire-weather interactions.^39^ Therefore, the 10-year experiments can be regarded as ten 1-year ensembles when studying the seasonal fluctuation for the fire-aerosol feedbacks.^74^^,^^75^

In both the control and sensitivity simulations, the same initial conditions after the long-term spinning up were used. The monthly climatological sea surface temperature and sea ice data averaged between 1981 and 2010 from the Met Office Hadley Centre (HadISST)^76^ were used as the boundary condition. The climatological lightning data is derived from the 3-hourly cloud-to-ground lighting in the NASA LIS/OTD grid product v2.2 fixed in 2000 (https://ghrc.nsstc.nasa.gov/uso/ds_docs/lis_climatology/lolrdc_dataset.html, last access: 30 October 2023). We obtained aerosol and nitrogen deposition rates from the monthly results of CESMv1.2 full chemistry simulations for 2000^77^ and used these data in land model simulations. The population density is from Gridded Population of the World version 3 fixed in 2000.^78^ The greenhouse gas (GHG) concentration is also fixed at the level of the year 2000 (367.0 ppmv CO_2_; 1760.0 ppbv CH_4_; 316.0 ppbv N_2_O). The GHG concentrations are imported from CAM5 to CLM4.5, which in return calculated the carbon flux and transports the net ecosystem exchange (NEE, in unit of g C m^-2^ s^-1^) back to CAM5. Anthropogenic emissions from the Fifth Assessment Report of the Intergovernmental Panel on Climate Change (IPCC AR5)^79^ in 2000 were used for non-fire emissions. The dimethyl sulfide (DMS) and volcanic sulfur emissions were from the prescribed Aerosol Inter-Comparison project (AeroCom) emission dataset. For land use and land cover change data, we applied the data for the year 2000 from Land-Use History A (LUHa) product version 1,^80^ which is then affected by post-fire activities in the model.^39^

The 10-year simulations are carried out with prescribed climatological sea ice data and sea surface temperature. Both ensemble simulations share the same initial conditions after a hundred-year spinning-up of CLM-RESFire,^39^ ensuring the attainment of a steady state with regards to land surface type alterations. Therefore the variations of the 10-year simulations reflects largely the timescale of the internal variability of the atmosphere (without atmosphere-land-ocean feedback). For these reasons, we treat the 10-year simulation as an ensemble of 10 1-year simulations as in previous studies.^1^^,^^61^^,^^62^ The ensemble means are therefore averages of the 10 1-year simulations.

CESM simulations have been extensively evaluated such as aerosol simulations,^64^^,^^81^ long-term temperature trend simulations,^82^ CESM performance in CMIP3, 5 and 6,^83^ CMIP6- Aerosol Chemistry Model Intercomparison Project (AerChemMIP),^84^ and the Atmospheric Chemistry and Climate Model Intercomparison Project (ACCMIP).^85^ To evaluate the CESM-RESFire model, A 20-year simulation was conducted^39^ with the atmosphere forced by Climatic Research Unit and National Centers for Environmental Prediction (CRUNCEP), which is a dataset combining observation and reanalysis data, including fire weathers including global surface temperature, humidity, wind, precipitation, and solar radiation. When forced by CRUNCEP and including both natural and anthropogenic constraints, the burned fraction simulated by CESM-RESFire had a high correlation (r=0.75) with the GFED fire inventory. Carbon emissions, interannual fire emission trend and FRP were also well evaluated in this previous study. In this study, we evaluated the model simulation of burned area in Africa by comparing to GFED4.1s^13^^,^^42^^,^^43^ (https://www.globalfiredata.org/, last access: 19 August 2020) and Fire Inventory from NCAR (FINN v2.5).^86^ The CESM-RESFire model simulations shown in Figures 1A and S6A well caught the spatial patterns and the seasonal variations shown in GFED and FINN (Figure S7). The CESM-RESFire simulated global burned area is 464 Mha yr^-1^ globally and 311 Mha yr^-1^ in Africa with fire aerosol feedback, which is comparable to the annual mean GFED4.1s burned area from 2001 to 2010 (485.4 Mha yr^-1^ globally and 333.5 Mha yr^-1^ in Africa) and the annual mean FINN v2.5 burned area from 2002 to 2011 (688.5 Mha yr^-1^ globally and 397.9 Mha yr^-1^ in Africa). The CESM-RESFire simulated burned area with no fire aerosol feedback is 1% lower (458 Mha yr^-1^) globally and 9% lower (283 Mha yr^-1^) in Africa, comparing with the simulations with fire feedback. This evaluation indicates that including the fire feedback improved model’s performance on simulating burned area.

CESM-RESFire coupled CAM5 as the atmospheric module, including the three-mode aerosol module (MAM-3),^64^ the Morrison-Gettelman cloud microphysics^71^^,^^72^ and cloud macrophysics.^70^ Previous studies evaluated the relationship between AOD and precipitation rate simulated in CESM and found good agreement with the satellite observations.^87^ The CESM-CAM5 model has also been used to assess aerosol-climate interactions, demonstrating strong performance in the simulations aerosol optical depth (AOD), cloud fraction, precipitation, and shortwave radiative flux.^88^ In this study, we evaluated both aerosol information (AOD) and meteorology (temperature, precipitation, cloud liquid amount) using observations and reanalysis data. We evaluated the model results using observation datasets including the satellite AOD data from MODIS Aqua level 3 monthly product^89^ in Figure S8. The model simulation agrees with MODIS AOD observations in terms of the African fire regions and the fire aerosol transport to the remote ocean. We compared the model simulated temperature and cloud liquid amount data with the National Centers for Environmental Prediction NCEP Climate Forecast System Reanalysis (CFSR) monthly products averaged from 2001 to 2010.^90^ The simulated zonal mean surface atmosphere temperature (Figure S9) and temperature profiles (Figure S10) agree will with CFSR in terms of the meridional distribution, although the model underestimated surface air temperature by ∼2 K over the equatorial Africa. In Figure S11, we compared the cloud liquid amount between the model simulations and CFSR. The simulation results are similar to CFSR data in the seasonal mean and meridional pattern of cloud distribution, but the simulation underestimated the cloud amount above 4 km, indicating a potential model bias of aerosol wet scavenging in deep convection.^91^^,^^92^ We also compared the zonal mean precipitation in Africa between the model simulations and Global Precipitation Climatology Project Version 2.3 (GPCP v2.3) produced 5 under the NOAA Climate Data Record (CDR)^93^ from 2001 to 2010 (Figure S12). The model distribution of the zonal mean precipitation in Africa is similar to GPCP data. The equatorial precipitation from model is overestimated by 8% - 15% in DJF, MAM and SON, but the bias is within the interannual variability. In DJF when the fire aerosol effect on precipitation reduction is large, the simulation with fire feedback agrees better with GPCP than the simulation without fire aerosols. More global evaluations for the CESM-RESFire model and the evaluations with ground based AOD measurements from level 2.0 Aerosol Robotic Network (AERONET)^94^ and the carbon budget with MODIS primary production products^95^^,^^96^ are described in Zou et al. (2019).^39^
