# Supplementary material for: Positive feedback to regional climate enhances African wildfires
Source: iScience. 2023 Nov 23;26(12):108533. doi: 10.1016/j.isci.2023.108533 (PMC10730378; doi:10.1016/j.isci.2023.108533)
Supplement: Document S1. Figures S1–S12 [file mmc1.pdf]

iScience, Volume *26*

Supplemental information

Positive feedback to regional climate enhances African wildfires

Aoxing Zhang, Yuhang Wang, and Yufei Zou

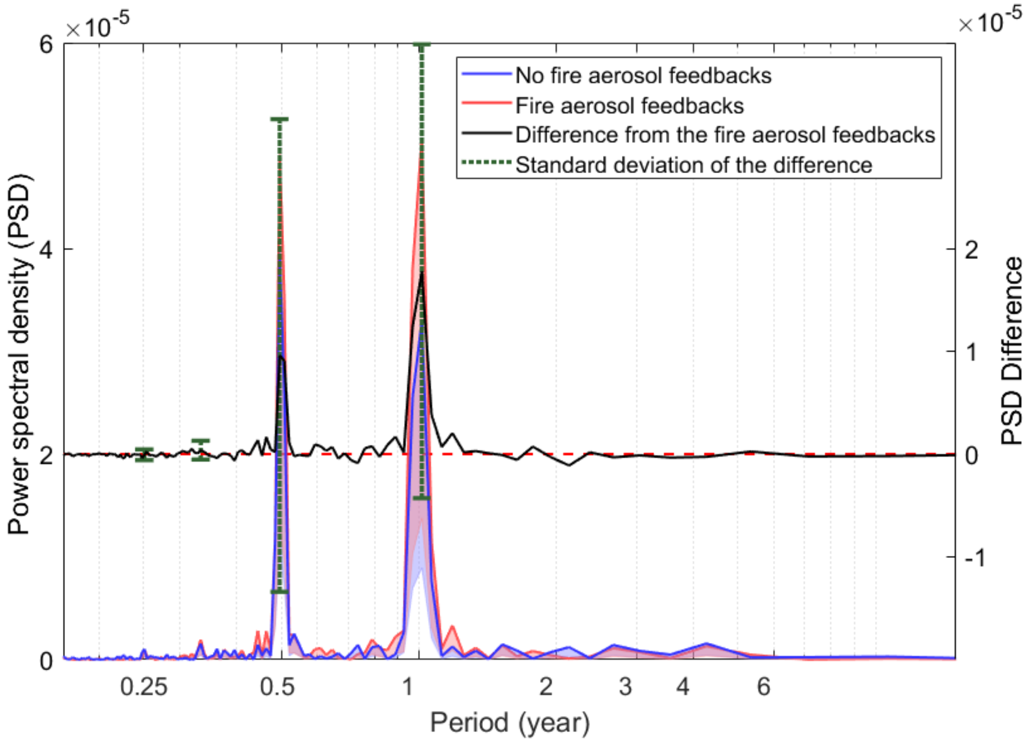

Figure S1. PSDs for monthly burned area with (shown in red) and without (shown in blue) fire aerosol feedbacks over Africa, related to the STAR Methods. The PSD difference due to fire aerosol feedbacks is shown in black. The color shaded area is the lower 95th percentile confidence level of the PSD with fire aerosol feedbacks. The dark green error bars represent the standard deviation of the PSD difference at periods of 3, 4, 6 and 12 months.

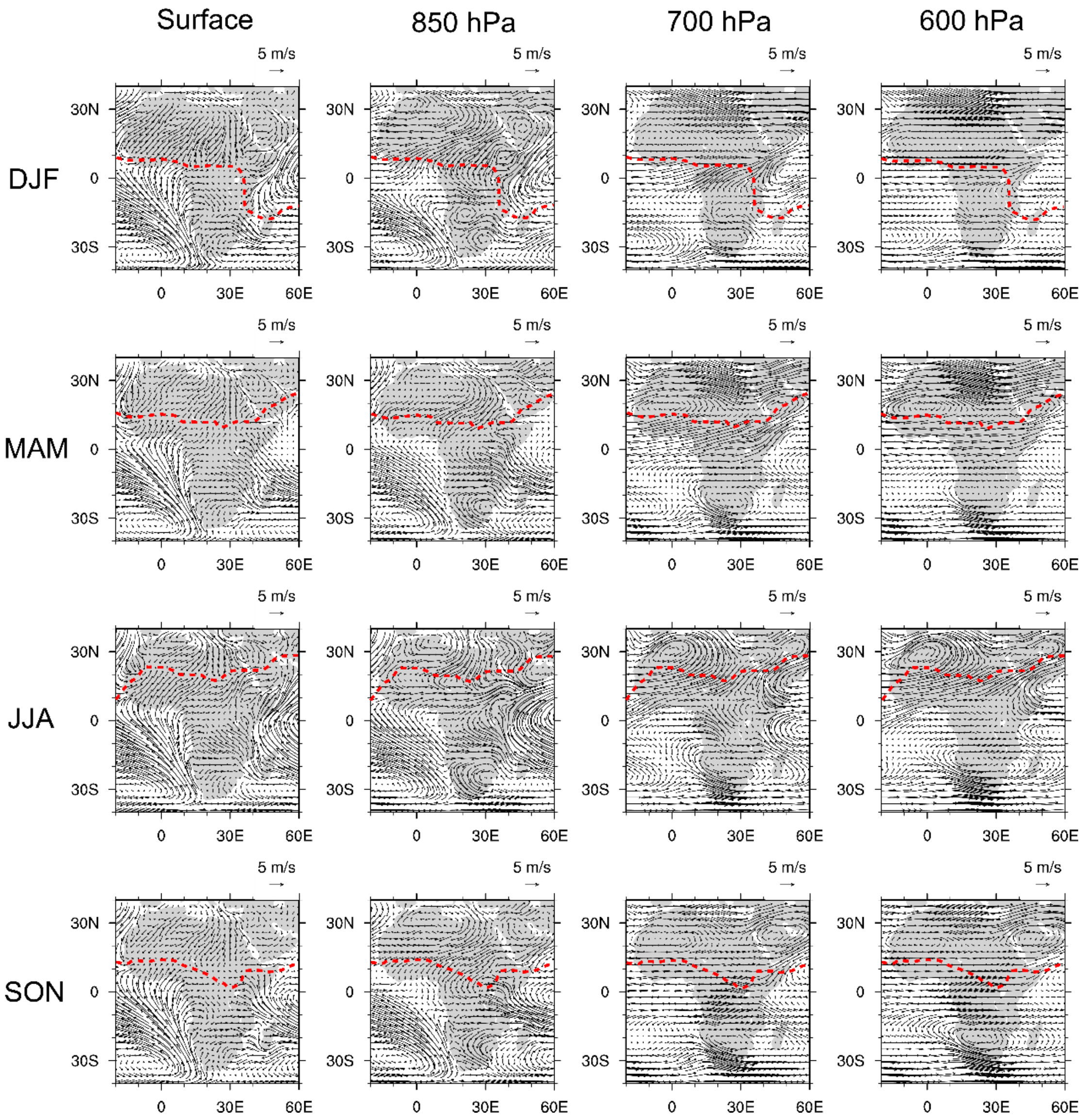

Figure S2. The seasonal mean wind direction and speed simulated in CESM_RESFire, related to the STAR Methods. Each panel represents a seasonal mean simulated wind direction and speed in DJF, MAM, JJA and SON, and at the levels of surface, 850 hPa, 700 hPa and 600 hPa. The reference vector of 5 m/s is included at the top right corner of each panel. The red dash lines are the ITCZ location determined by the surface wind convergence.

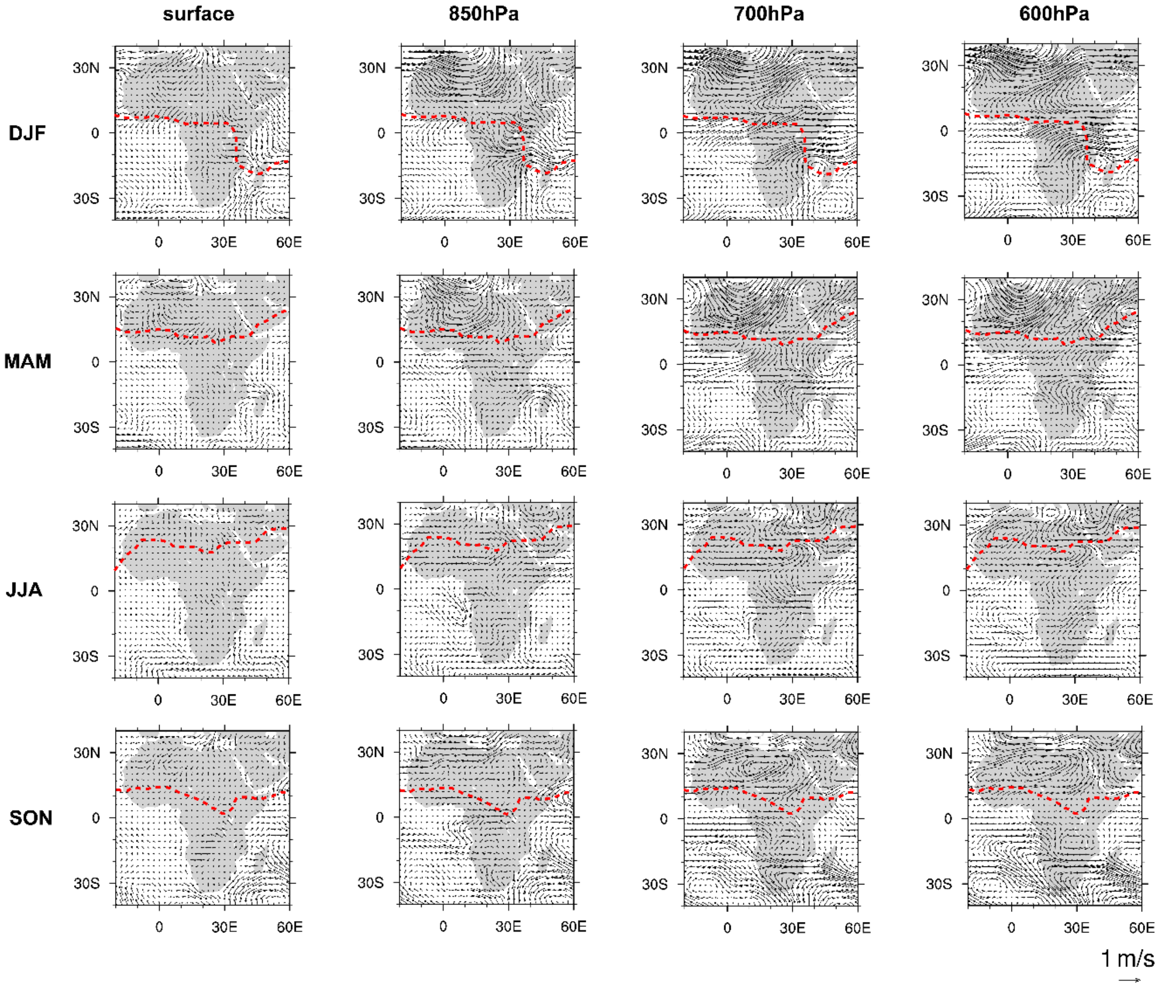

Figure S3. The fire-induced seasonal mean wind direction and speed changes simulated in CESM_RESFire, related to the STAR Methods. Each panel represents a seasonal mean wind direction and speed changes between the CESM_RESFire simulations with and without fire aerosol feedbacks, in DJF, MAM, JJA and SON, and at the levels of surface, 850 hPa, 700 hPa and 600 hPa. The reference vector of 5 m/s is included at the top right corner of each panel. The red dash lines are the ITCZ location determined by the surface wind convergence.

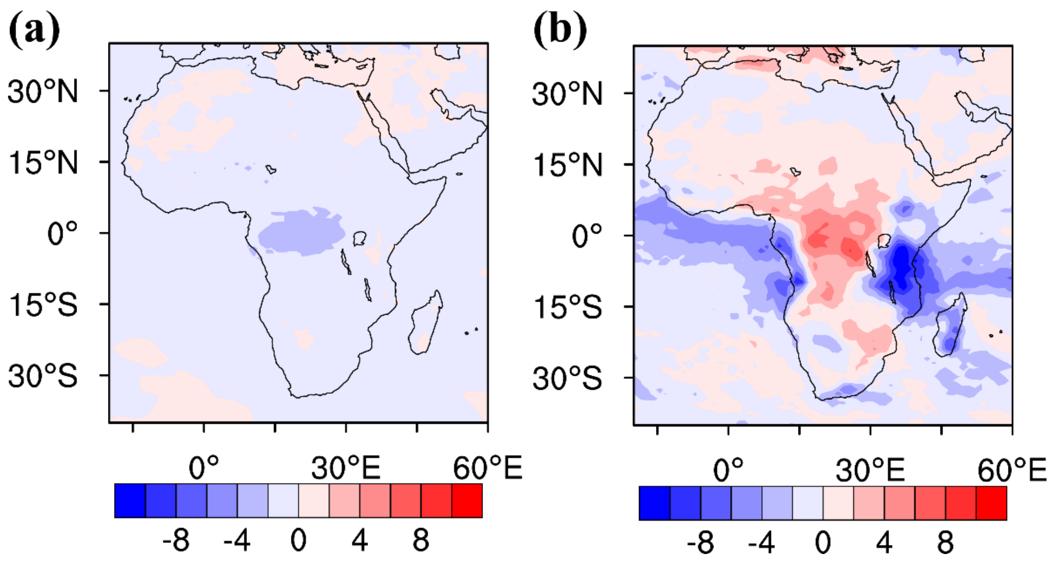

Figure S4. The annual mean fire-induced aerosol radiative effects at the top of the atmosphere, related to the STAR Methods. The direct aerosol radiative effect (unit: W m^{-2}) in **(a)** is much less than that from aerosol-cloud interactions in **(b)**.

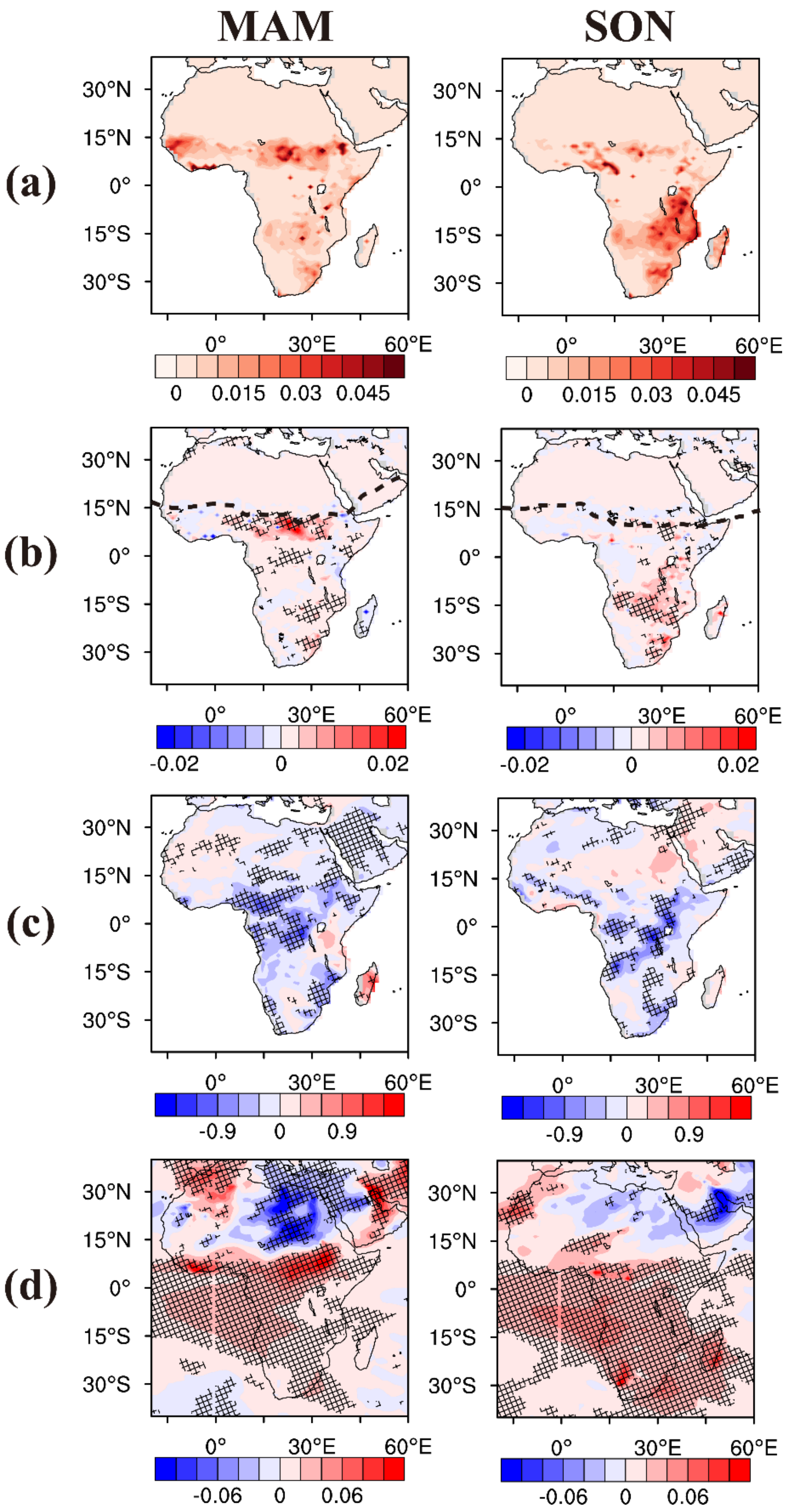

Figure S5. Same as Figure 1 but for MAM and SON, related to Figure 1.

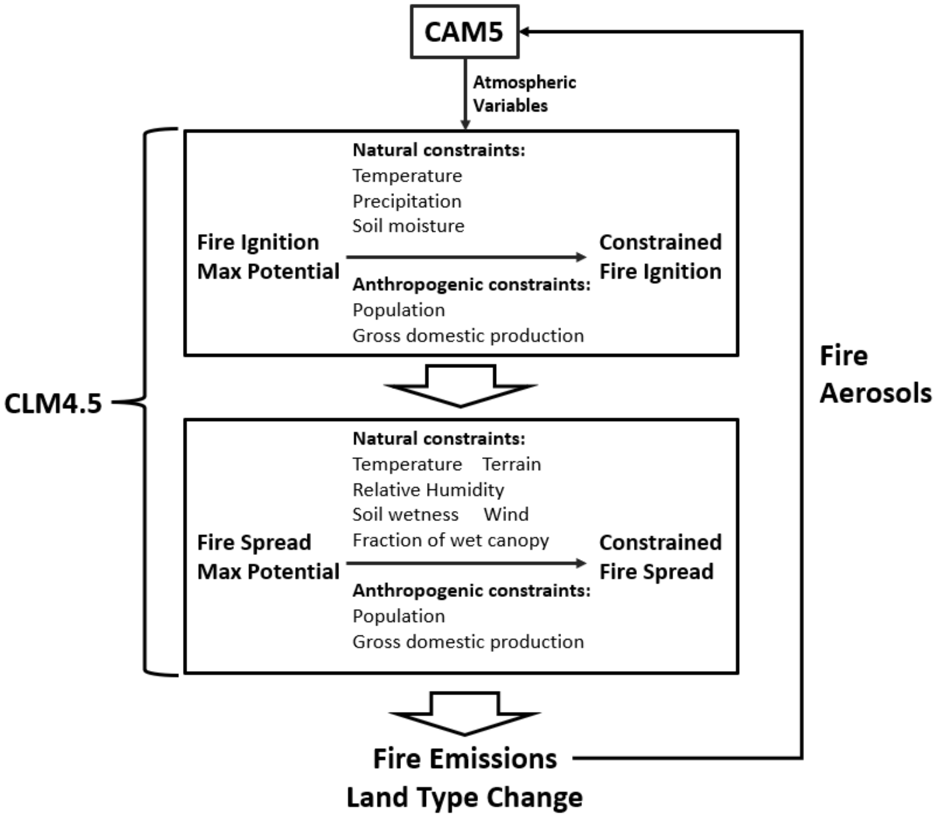

Figure S6. The schematic diagram describing the two-way coupled CESM-RESFire model, related to the STAR Methods.

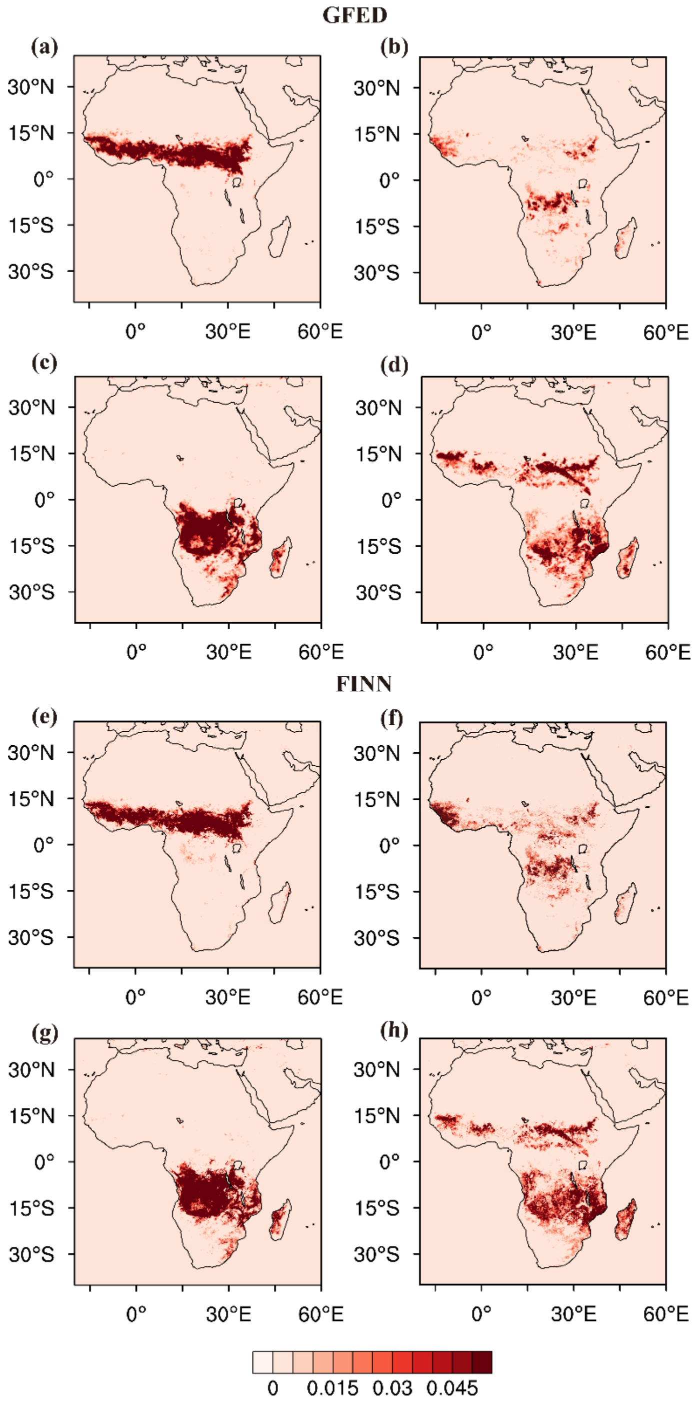

Figure S7. Seasonal mean burned fraction from different emission inventories, related to the STAR Methods. (a)-(d) represents the burned fraction from 2001-2010 GFEDv4.1s emission inventory in **(a)** DJF, **(b)** MAM, **(c)** JJA, **(d)** SON. **(e)-(h)** represents the burned fraction and from 2002-2011 FINNv2.5 in **(e)** DJF, **(f)** MAM, **(g)** JJA, **(h)** SON.

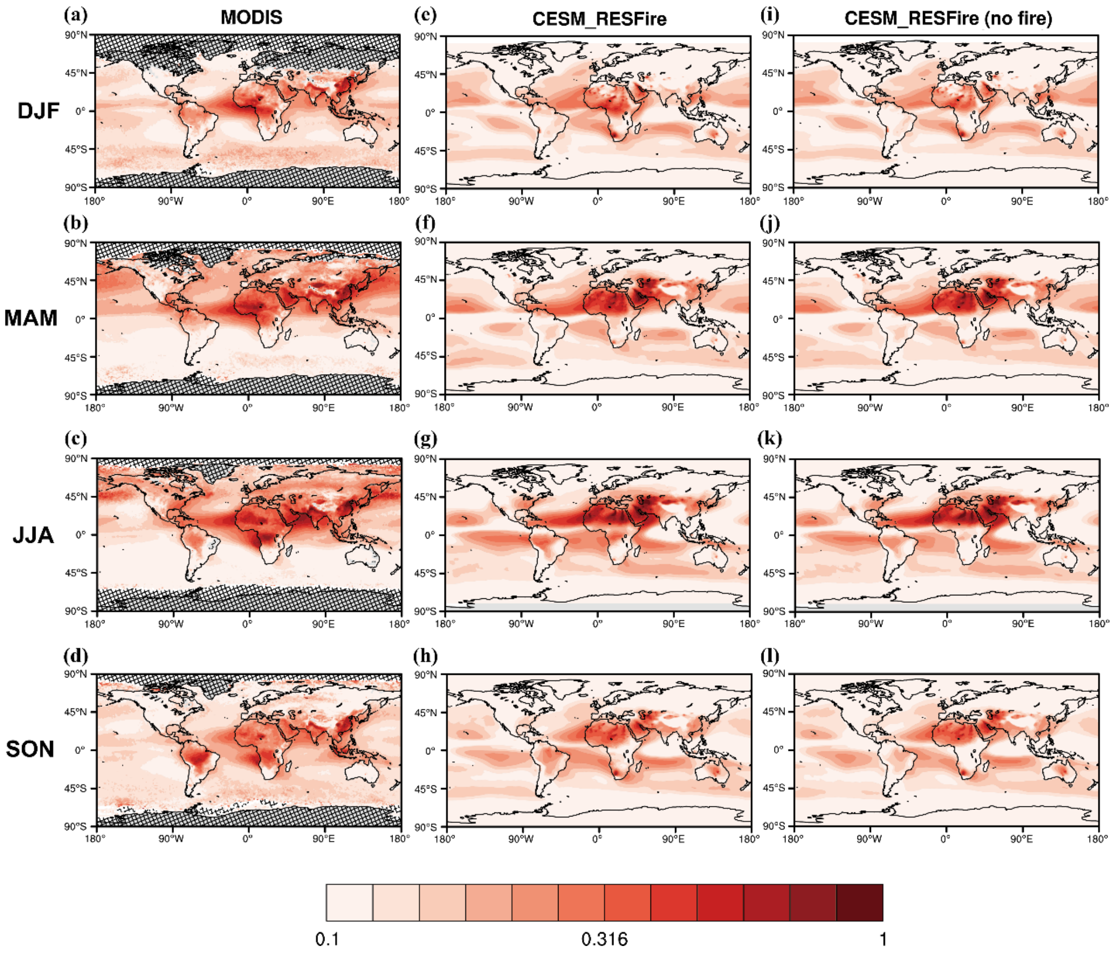

Figure S8. The comparison of AOD between MODIS and CESM_RESFire simulations, related to the STAR Methods. (a) - (d) The seasonal mean AOD global distributions from MODIS satellite product from 2003 to 2012. **(e) - (h)** The seasonal mean AOD global distributions from 10-year CESM_RESFire simulations with fire aerosol feedbacks. **(i) – (l)** The seasonal mean AOD global distributions from 10-year CESM_RESFire simulations with no fire aerosols. The seasonal means are averaged in **(a, e, i)** DJF, **(b, f, j)** MAM, **(c, g, k)** JJA and **(d, h, l)** SON. In **(a) - (d)**, the missing data are shaded.

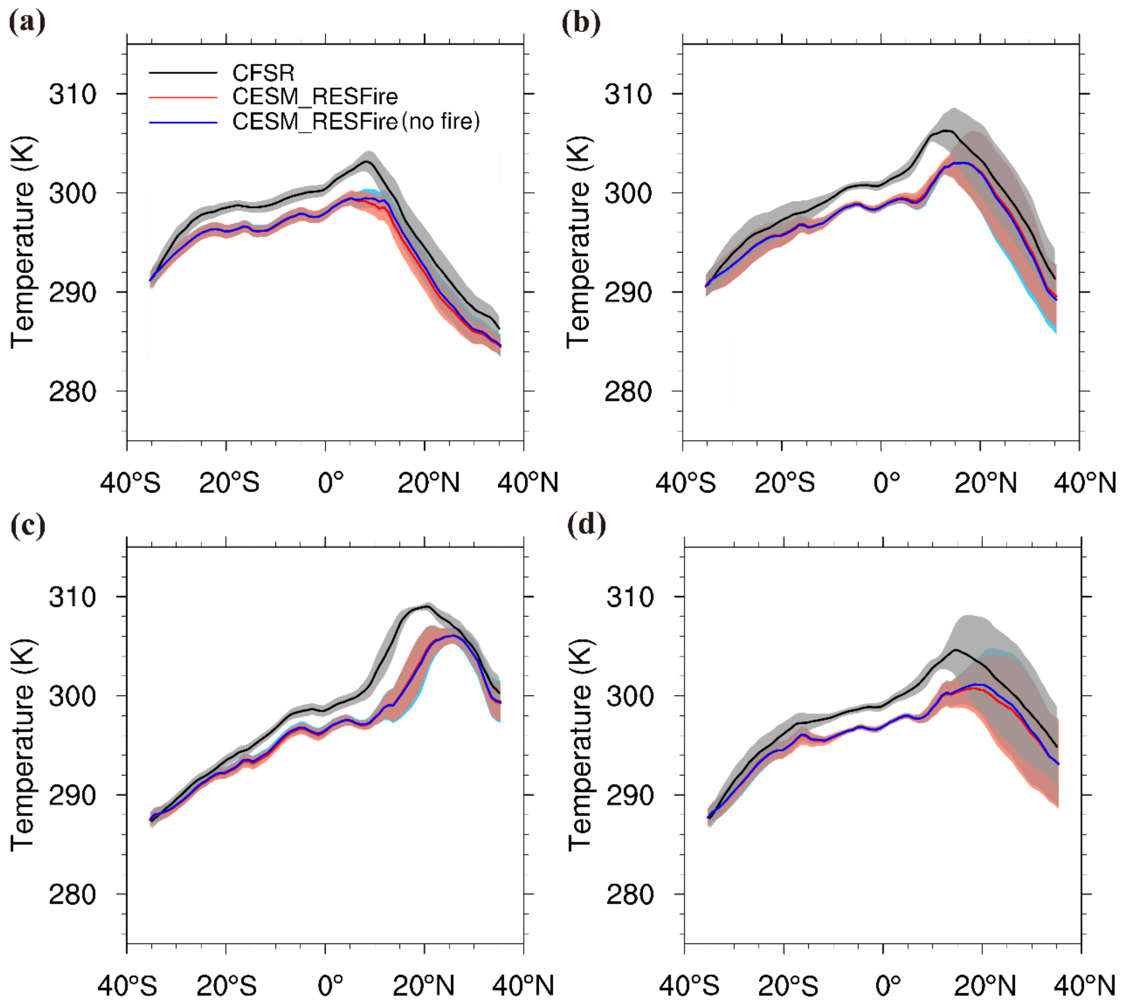

Figure S9. The comparison of zonal mean surface temperatures in Africa, related to the STAR Methods. Zonal mean surface temperatures (unit: K) averaged between 20° W and 50° E are compared between CFSR in 2001-2010 (black) and CESM_RESFire simulations with (red) and without (blue) fire aerosol feedbacks in **(a)** DJF, **(b)** MAM, **(c)** JJA and **(d)** SON. Uncertainties are shaded in the corresponded colors.

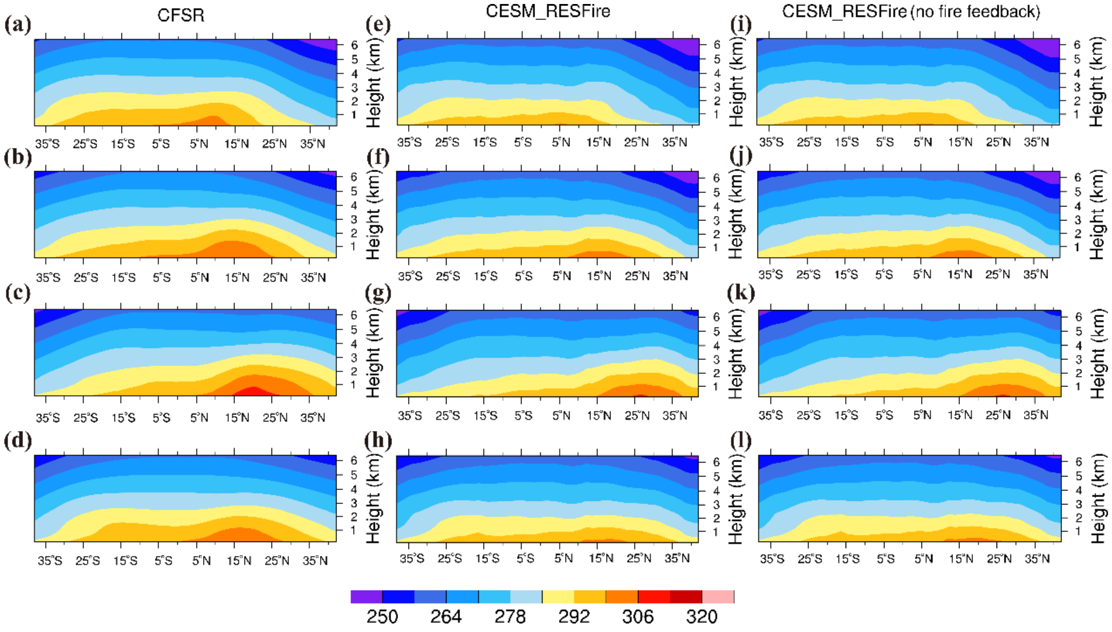

Figure S10. The comparison of zonal mean temperatures in Africa, related to the STAR Methods. Zonal seasonal mean temperature under 6 km (unit: K) averaged between 20° W and 50° E are compared between CFSR in 2001-2010 **(a) - (d)** and CESM_RESFire simulations with **(e) - (h)** and without **(i) - (l)** fire aerosol feedbacks in **(a, e, i)** DJF, **(b, f, j)** MAM, **(c, g, k)**, JJA and **(d, h, l)** SON.

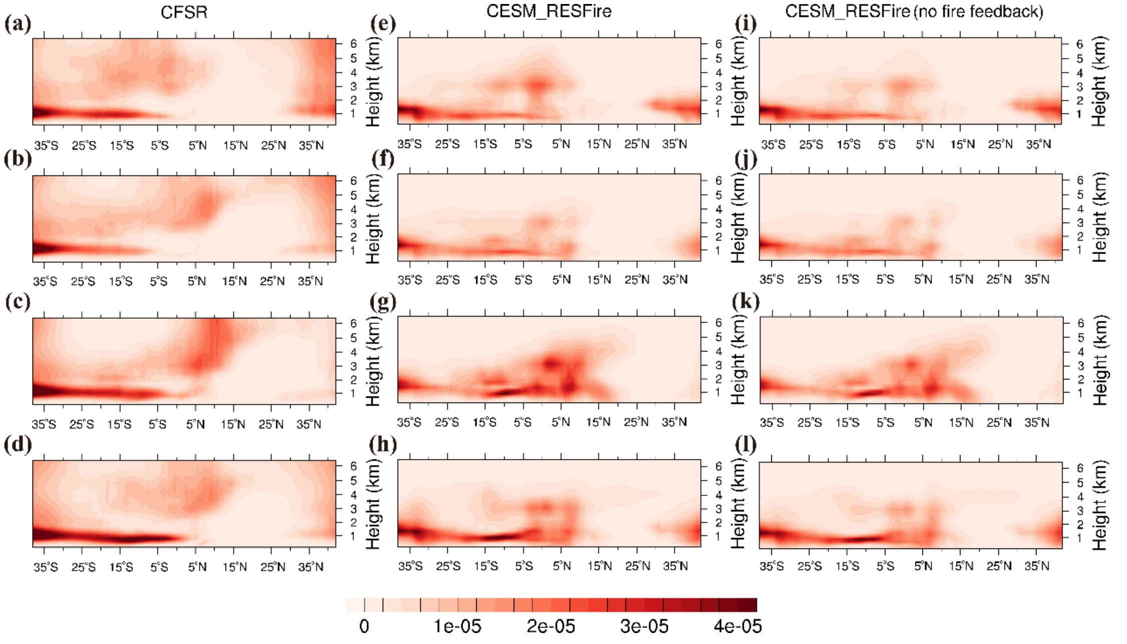

Figure S11. The comparison of zonal mean cloud liquid amounts in Africa, related to the STAR Methods. Zonal mean cloud liquid amounts (unit: kg kg^{-1}) averaged between 20° W and 50° E are compared between and CESM_RESFire simulations with fire aerosol feedbacks **(a) - (d)** and CFSR in 2001-2010 **(e) - (h)** in **(a, e)** DJF, **(b, f)** MAM, **(c, g)** JJA and **(d, h)** SON.

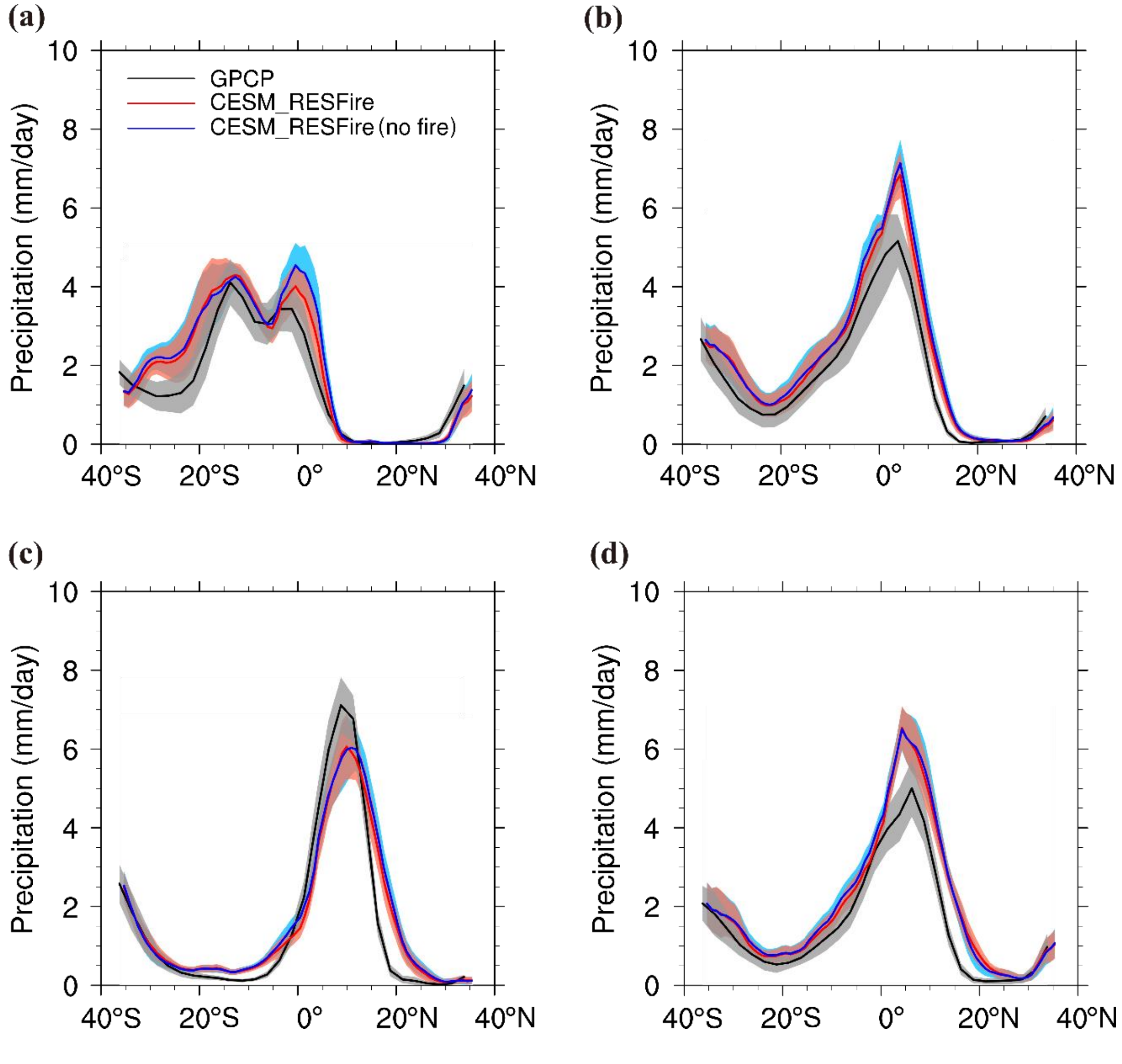

Figure S12. The comparison of zonal mean precipitations in Africa, related to the STAR Methods. Zonal mean precipitations (unit: mm day^{-1}) averaged between 20° W and 50° E are compared among GPCP in 2001-2010 (black) and CESM_RESFire simulations with (red) and without (blue) fire aerosol feedbacks in **(a)** DJF, **(b)** MAM, **(c)** JJA and **(d)** SON. Standard deviations of the seasonal mean precipitation are shaded in the corresponding colors.
